# UCP2 Deficiency Helps to Restrict the Pathogenesis of Experimental Cutaneous and Visceral Leishmaniosis in Mice

**DOI:** 10.1371/journal.pntd.0002077

**Published:** 2013-02-21

**Authors:** Javier Carrión, M. Angeles Abengozar, María Fernández-Reyes, Carlos Sánchez-Martín, Eduardo Rial, Gustavo Domínguez-Bernal, M. Mar González-Barroso

**Affiliations:** 1 Department of Animal Health, Veterinary Faculty, Complutense University of Madrid, Madrid, Spain; 2 Department of Chemical and Physical Biology, Centre for Biological Research CIB-CSIC, Madrid, Spain; 3 Department of Cellular and Molecular Medicine, Centre for Biological Research CIB-CSIC, Madrid, Spain; U.S. Food and Drug Administration, United States of America

## Abstract

**Background:**

Uncoupling protein 2 (UCP2) is a mitochondrial transporter that has been shown to lower the production of reactive oxygen species (ROS). Intracellular pathogens such as *Leishmania* upregulate UCP2 and thereby suppress ROS production in infected host tissues, allowing the multiplication of parasites within murine phagocytes. This makes host UCP2 and ROS production potential targets in the development of antileishmanial therapies. Here we explore how UCP2 affects the outcome of cutaneous leishmaniosis (CL) and visceral leishmaniosis (VL) in wild-type (WT) C57BL/6 mice and in C57BL/6 mice lacking the UCP2 gene (UCP2KO).

**Methodology and Findings:**

To investigate the effects of host UCP2 deficiency on *Leishmania* infection, we evaluated parasite loads and cytokine production in target organs. Parasite loads were significantly lower in infected UCP2KO mice than in infected WT mice. We also found that UCP2KO mice produced significantly more interferon-γ (IFN-γ), IL-17 and IL-13 than WT mice (*P*<0.05), suggesting that UCP2KO mice are resistant to *Leishmania* infection.

**Conclusions:**

In this way, UCP2KO mice were better able than their WT counterparts to overcome *L. major* and *L. infantum* infections. These findings suggest that upregulating host ROS levels, perhaps by inhibiting UPC2, may be an effective approach to preventing leishmaniosis.

## Author Summary

The leishmaniases comprise a group of diseases caused by infection by several species of intracellular protozoan parasites of the genus *Leishmania*, which are transmitted by the bite of an infected sandfly. The leishmaniases represent a global public health problem, affecting an estimated 12 million people around the world and ranging from self-healing skin lesions to potentially fatal systemic infections. Here, we use mouse models of CL and VL to investigate the effect of a host gene called UCP2. Uncoupling protein 2 (UCP2) is a mitochondrial carrier expressed in a wide variety of tissues, including white adipose tissue, skeletal muscle and the immune system. Intracellular pathogens such as *Leishmania* upregulate UCP2 and may weaken the immune system. Consequently, parasites survive and multiply within mouse macrophages. We demonstrate here that mice lacking the UCP2 gene are better able than WT mice to control both CL and VL. Infection was analyzed *in vivo* by measurement of footpad swelling, quantification of parasite load and assays for the production of cytokines and *Leishmania*-specific antibodies. These findings could have important implications in designing an effective approach to preventing leishmaniosis.

## Introduction

UCP2 is a mitochondrial carrier expressed in a wide variety of tissues, including white adipose tissue, skeletal muscle and the immune system [1]. UCP2 activity presumably lowers the efficiency of oxidative phosphorylation by increasing the membrane proton conductance. This effect would result in an increased rate of respiration leading to a downregulation of the mitochondrial production of ROS. This uncoupling activity is widely considered as an additional element of the cellular antioxidant defence [2]–[4]. In fact, UCP2 is upregulated in many pathological processes in which ROS play an important role in the development of the disease (atherosclerosis, cancer, chronic inflammation, etc.) [2]. In addition, UCP2 has been postulated to help regulation of insulin secretion by pancreatic β-cells [5]. Indeed, loss-of-function mutations of UCP2 have been identified in patients suffering from congenital hyperinsulinism. It has been shown that UCP2 modulates glucose-stimulated insulin secretion by reducing mitochondrial ROS [6].

By decreasing ROS production, UCP2 may weaken the immune system when faced with attack by bacterial and parasitic pathogens. Mice lacking UCP2 showed higher survival rates than WT mice following experimental infection with *Listeria monocytogenes* [7] or *Toxoplasma gondii* [8]. A recent *in vitro* study demonstrated that *Leishmania donovani* upregulates UCP2 and thereby suppresses mitochondrial ROS production, leading to increased production of anti-inflammatory cytokines and parasite survival inside murine macrophages [9]. The same authors found that silencing UCP2 *in vivo* in splenic tissue of BALB/c mice 3 days prior to *L. donovani* infection stimulates ROS production, shifts the balance of pro- vs. anti-inflammatory cytokines towards the pro-inflammatory phenotype and reduces splenic parasite burden after 4–6 weeks of infection. These findings raise the possibility of targeting host UCP2 activity and ROS production as a strategy to develop effective therapies against leishmaniosis. To make this work possible, the role that host UCP2 plays in the host immune response to *Leishmania* infection should first be investigated *in vivo*.

The leishmaniases comprise a group of diseases caused by infection by several species of intracellular protozoan parasites of the genus *Leishmania*, which are transmitted by the bite of an infected female phlebotomine sandfly [10]. Infections with *L. major* and *L. infantum* are distinguished by clinical manifestations ranging from localized skin ulcers at the site of the sandfly bite (CL), to potentially lethal visceral disease (VL), respectively. The leishmaniases represent a global public health problem, affecting an estimated 12 million people around the world. Indeed, 1.5 million new cases of CL and 0.5 million new cases of VL are reported in humans each year [11]. Current research has focused on the development of diagnostic methods, drugs and vaccines. Unfortunately, despite considerable progress, treatments are toxic and expensive [12] and no vaccines are available for any form of human leishmaniosis [13], [14]. The leishmaniases are classified as neglected tropical diseases (NTDs), which still require improved treatment strategies and new prophylactic vaccines [15].

To investigate the role of UCP2 in host immune defense against intracellular pathogens, we explored in detail the outcomes of CL and VL due to *L. major* and *L. infantum* infection of WT C57BL/6 mice and UCP2 knockout (UCP2KO) C57BL/6 mice. Infection was analyzed *in vivo* by measurement of footpad swelling, quantification of parasite load and assays for the production of cytokines and *Leishmania*-specific antibodies. Better understanding of the role of host UCP2 in the immune response to *Leishmania* parasites should help develop more effective strategies to control disease.

## Materials and Methods

### Ethics statement

The animal research described in this manuscript complied with Spanish (Ley 32/2007) and European Union legislation (2010/63/UE). The protocols used were approved by the Animal Care Committee of Complutense University of Madrid.

### Animals

C57BL/6 mice were purchased from Harlan Interfauna Ibérica (Barcelona, Spain). UCP2KO mice with a C57BL/6 background were obtained from JAX mice (The Jackson Laboratory, Bar Harbor, USA). In infection experiments, mice were matched for age and sex (females, 8–9 weeks old).

### Parasites and preparation of soluble antigen

*L. major* parasites (clone V1: MHOM/IL/80/Friedlin) and *L. infantum* parasites (MCAN/ES/96/BCN/150, MON-1) were cultured at 26°C in Schneider's medium (Sigma-Aldrich, Madrid, Spain) containing 20% heat-inactivated fetal calf serum (FCS, Sigma-Aldrich), streptomycin and penicillin. Soluble *Leishmania* antigen (SLA) was prepared from stationary cultures of *L. major* promastigotes as previously described [16]. *Leishmania pifanoi* axenic amastigote strain MHOM/VE/60/Ltrod (kindly provided by A. A. Pan, The Johns Hopkins School of Hygiene and Public Health, Baltimore, Maryland 21205, USA) was cultured at 32°C in M199 medium supplemented with 20% heat-inactivated FCS, 2.5% (w/v) trypticase peptone, 13.8 mM d-glucose, 76 µM hemin, and 48 µg/mL gentamicin sulfate at pH 7.2 [17].

### Preparation of peritoneal macrophages

Three days after intraperitoneal injection of 1 ml of 4% thioglycollate (Difco Laboratories, Detroit, MI), cells were obtained by peritoneal lavage with cold phosphate-buffered saline (PBS). Then cells were washed and plated as needed for each experiment in RPMI medium containing 10% heat-inactivated FCS.

### **C**arboxyfluorescein succinimidyl ester (CFSE) labeling of *L. pifanoi* and *in vitro* infection

*L. pifanoi* amastigotes were labeled using the fluorescent dye CFSE (Invitrogen, San Diego, CA). Briefly, parasites were incubated in PBS at 4×10^6^ parasites/ml CFSE (0.4 µg/ml) for 30 min at 32°C. This process does not affect parasite multiplication [18]. To evaluate intracellular killing of *L. pifanoi* amastigotes we seeded peritoneal macrophages onto LabTek culture chamber incubation slides (Thermo Scientific, Madrid, Spain) at 5×10^4^ per chamber. On the following day, adherent macrophages were incubated for 4 h with *L. pifanoi* amastigotes in the ratio 4∶1. Then cells were washed until all free parasites were removed. After 24 h, confocal images were acquired using a confocal laser scanning microscope (Leica TCS – SP2 ABOS), and the infection rate was assessed by counting both the number of infected macrophages and the number of parasites per infected macrophage.

### Fluorescent detection of mitochondrial ROS

ROS production in peritoneal macrophages isolated from UCP2KO and WT mice was estimated from the rate of fluorescence increase of the cell-permeant redox-sensitive fluorescent indicators dihydroethidium (DHE) and 2′,7′-dichlorodihydrofluorescein diacetate (DCFDA). Cells (25×10^3^ per well) were seeded in 96-well plates in RPMI containing 10% FCS. When required, 4 h before the determination, macrophages were infected with *L. pifanoi* at a ratio 3∶1 and incubated at 32°C in RPMI without FCS and phenol red. After the incubation, plates were transferred to a Varioskan Flash microplate reader and the fluorescent probe of interest added to each well. Fluorescence intensity was recorded for 50 min, with measurements every 2 min, and the maximum rate of fluorescence increase calculated after subtraction of the rate obtained in the absence of cells. The rate of hydrogen peroxide formation was estimated with the probe DCFDA (10 µM, excitation 485 nm, emission 535 nm) and the rate of superoxide formation with the probe DHE (50 µM, excitation 535 nm, emission 610 nm).

### NO release and IL-12 p40 production by uninfected and infected macrophages

To measure NO release, peritoneal macrophages were plated at 5×10^4^ per well in 96-well plates in the presence of 10 ng/ml recombinant mouse IFN-γ (Sigma-Aldrich). After 24 h, parasites were added and NO release was measured 24 h later using Griess reagent as described below. To assay interleukin-12 (IL-12), peritoneal macrophages were cultured at 2×10^5^ cells per well in 24-well plates and incubated in the presence of 10 ng/ml recombinant mouse IFN-γ for 24 h, as previously described [19]. Subsequently macrophages were stimulated with lipopolysaccharide (LPS, 20 ng/ml) and infected with *Leishmania* parasites (*L. pifanoi*, *L. major* and *L.infantum*, parasite/cell ratio = 5∶1). After 24 h, culture supernatants were harvested and IL-12 p40 secretion was assayed by ELISA (BD Biosciences, Madrid, Spain), according to the manufacturer's instructions.

### Generation of bone marrow-derived murine dendritic cells (DCs)

We obtained bone marrow stem cell progenitors from the femurs and tibiae of naïve C57BL/6 mice and cultured the cells in the presence of 20 ng/ml murine granulocyte macrophage colony-stimulating factor (GM-CSF; PeproTech, London, UK), as previously described [20]. We added fresh medium containing GM-CSF to the cell cultures every 3 days, generating many CD11c^+^ DCs largely free of granulocyte and monocyte contamination. On day 7, DCs were plated at 1×10^6^ cells/ml in 6-well plates and primed in the presence or absence of SLA (50 µg/ml). DCs were collected at 24 h after pulsing with SLA and used for *in vitro* stimulation of T cells as described below.

### *In vivo* infections

Experimental CL was initiated by subcutaneous injection of 5×10^3^ metacyclic *L. major* promastigotes in 30 µl PBS into the left footpad (at least 6 mice per group). Metacyclic forms had previously been isolated from stationary cultures by negative selection using peanut agglutinin (Vector Laboratories, Barcelona, Spain) [21]. Footpad thickness was measured weekly with a metric caliper. Draining lymph nodes (DLNs) and spleens from euthanized mice were removed after 4 and 12 weeks of infection and subjected to a limiting dilution assay.

Experimental VL was initiated by intravenous injection of 5×10^5^ *L. infantum* promastigotes in 100 µl PBS (at least 6 mice per group). Spleens and livers from euthanized mice were removed after 8 weeks of infection and subjected to a limiting dilution assay.

In addition, NO release, arginase activity, cytokine profile and humoral response were determined in both CL and VL experimental models, as described below.

### Limiting dilution assay

Parasite burdens in DLNs, spleens and livers were determined by limiting dilution culture [22]. Briefly, organs were harvested aseptically and homogenized in 1 ml of Schneider's medium (Sigma-Aldrich) containing 20% FCS, streptomycin and penicillin. Four-fold serial dilutions were carried out in 96-well culture plates and incubated at 26°C. After culturing for 10 days, the highest dilution yielding growth of viable *Leishmania* promastigotes was determined using a phase contrast microscope.

### Cytokine assay

Mice were infected as described above. At 4 weeks after *L. major* infection, or 8 weeks after *L. infantum* infection, mice were euthanized and single-cell suspensions of DLNs or spleens were prepared, respectively. Previously, bone marrow-derived DCs from naïve C57BL/6 mice had been generated and pulsed with SLA (50 µg/ml), as described above. T cells were washed, resuspended at a final concentration of 2×10^6^ per ml in complete Dulbecco's modified Eagle's medium (DMEM supplemented with 10% heat-inactivated FCS, 2 mM l-glutamine, 100 U/ml penicillin, and 100 µg/ml streptomycin), and plated at 1 ml/well in 24-well plates. T cells and DCs were mixed at a ratio of 5∶1 and cultured for 72 h. Culture supernatants were harvested and ELISA was used according to the manufacturer's instructions to assay secretion of the following mouse cytokines: IFN-γ (Diaclone, Madrid, Spain), IL-17 (R&D Systems, Madrid, Spain), IL-4 (eBioscience, Barcelona, Spain), IL-10 (Diaclone) and IL-13 (R&D Systems).

### NO assay

Concentration of nitrite, which is a byproduct of nitric oxide (NO) production, was measured in the culture supernatant after 72 h using the Griess assay as described [23]. Briefly, 100 µl of culture supernatant was mixed with an equal volume of Griess reagent (Sigma-Aldrich) and incubated at room temperature for 10 min. Absorbance was then measured at 540 nm and nitrite concentration calculated by comparison with a standard curve of serial dilutions of sodium nitrite.

### Arginase activity assay

After removing supernatants to measure NO release at 72 h, cells were incubated for 30 min in lysis buffer (0.1 M Tris-HCl, pH 7.5, 300 µM NaCl, 1 µM PMSF, 1% Triton X-100). Lysates were then assayed for intracellular arginase activity as previously described [24], [25]. One unit of enzyme activity was defined as the amount of enzyme that catalyzes the formation of 1 mmol of urea/min.

### Humoral immune response

*Leishmania*-specific antibodies were quantified by ELISA. Standard plates were coated overnight at 4°C with 100 µL of SLA (4 µg/mL) diluted in PBS. Then wells were washed with PBS supplemented with 0.05% (v/v) Tween-20 and blocked with 2% (w/v) bovine serum albumin (BSA) in PBS. Sera were serially diluted in order to determine the titer, which was defined as the inverse of the highest serum dilution factor giving an absorbance >0.2. Peroxidase-conjugated goat anti-mouse IgG1 and IgG2 were used as secondary antibodies (1∶2500 and 1∶500, respectively; SouthernBiotech, Madrid, Spain). After washing and adding peroxidase substrate (Ultra TMB-ELISA, ThermoScientific, Madrid, Spain), the reactions were stopped with 2 M sulfuric acid. Finally, sample absorbance was measured at 450 nm.

### Statistical analysis

Since measurements showed a standard normal distribution, Student's *t* test was used to evaluate the significance of differences between means of the experimental groups. Differences were considered significant when *P*<0.05. Statistical analyses were performed using SigmaPlot (version 12.2, Systat Software).

## Results

### *In vitro* studies of experimental leishmaniosis with peritoneal macrophages from UCP2KO and WT C57BL/6 mice

**Figure 1:**
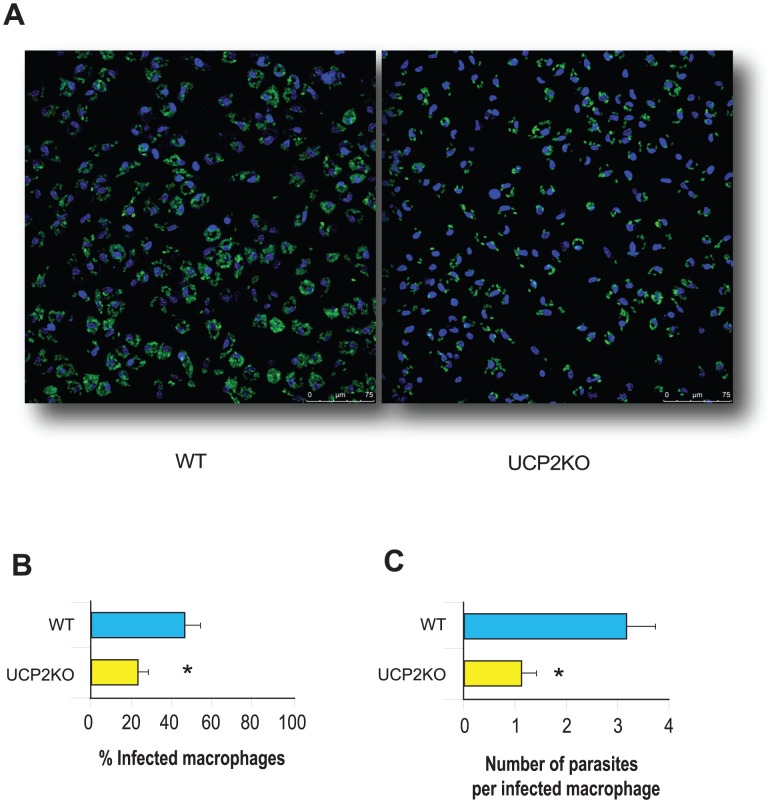
Intracellular killing of *L. pifanoi* amastigotes by macrophages. To determine anti-leishmanial activity we seeded peritoneal macrophages from WT and UCP2KO miceonto LabTek chamber slides at 5×10^4^ per chamber. After 4 h of infection with *L. pifanoi* amastigotes labelled with CFSE (ratio 4∶1), cells were washed until all free parasites were removed. (A) Confocal images were acquired 24 h later using a confocal laser scanning microscope, and the infection rate was assessed by counting both (B) the percentage of infected macrophages and (C) the number of parasites per infected macrophage. Asterisks indicate *P*<0.05 with respect to WT mice. (n = 6).

**Figure 2:**
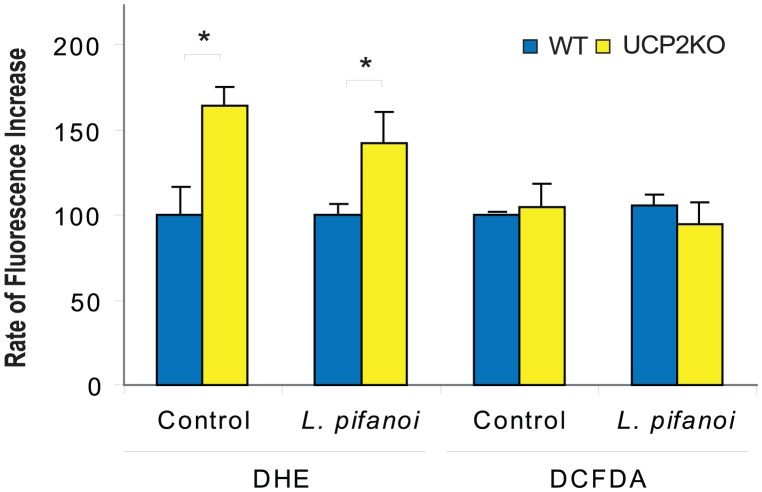
ROS production. Levels of ROS in peritoneal macrophages isolated from UCP2KO and WT mice were estimated from the rate of fluorescence increase of the cell-permeant redox-sensitive fluorescent indicators DHE and DCFDA. When required, 4 h before the determination, macrophages were infected with *L. pifanoi* at a ratio 3∶1. The rate of superoxide formation was estimated with the probe DHE (50 µM, excitation 535 nm, emission 610 nm) and the rate of hydrogen peroxide formation with the probe DCFDA (10 µM, excitation 485 nm, emission 535 nm). Asterisks indicate *P*<0.05 with respect to WT mice. (n = 6).

In this study, *in vitro* results will be reported primarily for the axenic *Leishmania pifanoi* amastigote line, whose similarity to the amastigotes isolated from infected macrophages has been exhaustively demonstrated [26]. To investigate the ability of macrophages from UCP2KO and WT mice to eliminate *L. pifanoi* amastigotes *in vitro*, we determined infection rates in macrophages. As demonstrated by confocal imaging (Fig. 1A), the percentage of infected macrophages and the number of parasites per macrophage after 48 h were found to be lower in UCP2KO macrophages than in WT ones (Fig. 1B–C). Our data showed that the rate of superoxide formation in macrophages from UCP2KO mice was higher than in those from WT mice. In contrast, hydrogen peroxide levels seemed to be identical and did not vary upon infection with *L. pifanoi* (Fig. 2).

Intracellular killing of *Leishmania* amastigotes by macrophages requires production of both IL-12 and NO [27]. Therefore we compared IL-12 and NO production in macrophages from UCP2KO and WT mice following *in vitro* infection with *L. pifanoi*, *L. major* or *L. infantum*. Peritoneal macrophages were primed with IFN-γ before the addition of LPS and parasites, as this is known to be required for considerable production of IL-12 by macrophages [28], [29]. We observed that *in vitro* infection with any of the *Leishmania* species did not suppress IL-12 p40 production. In addition, IL-12 release revealed no significant difference between peritoneal macrophages from UCP2KO and WT mice (Table 1). In contrast, *in vitro* infection with any of the three *Leishmania* species increased nitrite production in macrophages from UCP2KO mice but not in macrophages from WT mice (Table 2).

**Table 1:** IL-12 secretion by elicited macrophages from peritoneum from WT and UCP2KO mice.

IL- 12 p40 (pg/ml)					
	Control	Control	IFN-γ + LPS +	IFN-γ + LPS +	IFN-γ + LPS +
	Medium	IFN-γ + LPS	*L. pifanoi* 5∶1	*L. major* 5∶1	*L. infantum* 5∶1
**WT**	**25**±25	**273**±48	**243**±29	**246**±34	**244**±31
**UCP2KO**	**31**±25	**308**±34	**264**±21	**257**±28	**283**±28

Macrophages were primed with IFN-γ for 24 h and then challenged for another 24 h in the presence of LPS and parasites (ratio 5∶1). Controls with medium only, or IFN-γ and LPS were included. After 24 h, culture supernatants were harvested. Data are presented as mean ± S.D. (n = 6).

**Table 2:** Nitrite production.

Nitrites (µM)				
	Control	*L. pifanoi*	*L. major*	*L. infantum*
**WT**	12±2	13±2	14±2	15±2
**UCP2KO**	17±3	19±3 (*)	20±2 (*)	25±3 (*)

The NO production was indirectly determined by measuring nitrite level in the cultured supernatant by Griess reaction at 24 h after infection. Control: uninfected mice. Data are presented as mean ± S.D. (n = 6). Asterisks indicate *P*<0.05 with respect to WT macrophages.

### Course of experimental CL in UCP2KO and WT C57BL/6 mice

**Figure 3:**
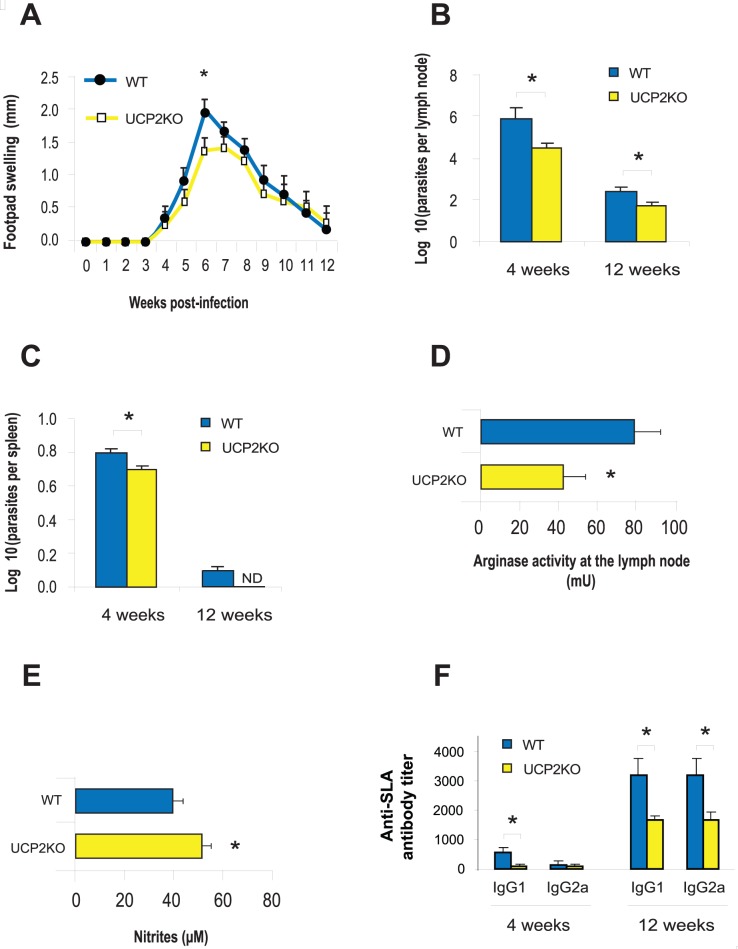
UCP2KO mice display enhanced resistance to CL. UCP2KO and WT C57BL/6 mice were challenged subcutaneously with 5×10^3^ *L. major* metacyclic promastigotes and the course of infection was evaluated. A. Mean diameter of induration (± S.D) in the footpad at various times after infection. B. Mean number of parasites per popliteal DLN (± S.D) at 4 and 12 weeks after infection. C. Mean number of parasites per spleen (± S.D) at 4 and 12 weeks after infection. D–E. Competition between arginase and iNOS (nitrite levels) enzymes in DLNs was assessed at 4 and 12 weeks after *L. major* infection. Single cell suspensions of DLNs from euthanized mice were cultured for 72 h in the presence of SLA-pulsed DCs. Arginase activity was measured in cell lysates and nitrite levels were measured in the culture supernatants. F. Anti-SLA antibody (IgG1 and IgG2a) titers in sera from WT and UCP2KO mice at 4 and 12 weeks after infection. Data are presented as mean ± S.D. (n = 6). N.D., parasites not detected. Asterisks indicate *P*<0.05 with respect to WT mice.

Disease progression in mice infected with *L. major* was evaluated by weekly monitoring of the appearance and extent of footpad swelling. Infection led to significantly smaller footpad swelling (after 6 weeks of infection) and lower parasite burden in DLNs in UCP2KO mice than in WT mice (Fig. 3A, 3B). Lesions ultimately resolved at the same time in both groups.

We also analyzed parasite loads in spleen in order to investigate systemic infection. We did not detect *L. major* parasites in spleens of UCP2KO mice after 12 weeks of infection (Fig. 3C), while 50% of WT mice had not yet eliminated the parasites by this time. It is important to point out that C57BL/6 mice are resistant to cutaneous *L. major* infection and develop small, self-resolving lesions [10].

### Competition between arginase and iNOS enzymes in phagocytic cells contributes to the outcome of *L. major* infection

To investigate whether the increased resistance of UCP2KO mice to *L. major* infection correlates with arginase and iNOS activity, we measured arginase enzymatic activity and levels of nitrite in the popliteal lymph nodes that were draining the infection site after 4 weeks of infection. Arginase activity in the draining popliteal lymph node was significantly lower in UCP2KO mice than in WT mice (Fig. 3D). As expected, arginase activity and NO production were undetectable in uninfected UCP2KO and WT mice (data not shown). We also measured iNOS activity indirectly by assaying nitrite production after stimulating DLN cells with SLA-pulsed DCs. Nitrite levels were significantly higher in DLN cells from UCP2KO mice than in DLN cells from WT mice (Fig. 3E).

### Humoral immune response and cytokine production in UCP2KO and WT C57BL/6 mice infected with *L. major*

The Th1/Th2 paradigm of resistance/susceptibility to intracellular infection is based largely on studies using *L. major*. Th1 cells secrete cytokines that promote cell-mediated immunity, while Th2 cells secrete cytokines like IL-4 that induce antibody production [30]. *Leishmania* is an intracellular pathogen that avoids the humoral defenses of the host immune system. Thus, anti-*Leishmania* IgG1 antibody production fails to protect against this parasite and contributes to disease progression [31]. To characterize the humoral immune response to *L. major* infection, we measured levels of anti-SLA IgG1 and IgG2a antibodies in sera from both groups of mice (Fig. 3F). *Leishmania*-specific IgG1 antibody levels were significantly lower in UCP2KO mice than in WT mice after 4 and 12 weeks of infection. Nevertheless, the mean Th1/Th2 humoral ratio after infection was similar between WT and UCP2KO mice.

In order to determine what type of immune response is elicited after *L. major* infection, we assayed IFN-γ, IL-17 and IL-4 production in popliteal DLNs after *in vitro* stimulation with SLA-pulsed DCs. After 4 weeks of infection, DLN cells of *L. major*-infected UCP2KO mice produced significantly lower levels of SLA-specific IL-4 than those of WT mice, but they produced significantly larger amounts of SLA-specific IFN-γ and IL-17 (Table 3).

**Table 3:** Cytokine production in mice infected with *L. major* at 4 weeks after infection.

pg/ml			
	IFN-γ	IL- 17	IL- 4
**WT**	737±150	238±50	85±10
**UCP2KO**	1162±150 (*)	442±50 (*)	43±10 (*)

Data are presented as mean ± S.D. (n = 6). Asterisks indicate *P*<0.05 with respect to WT mice.

### Outcome of *L. infantum* infection in UCP2KO and WT C57BL/6 mice

**Figure 4:**
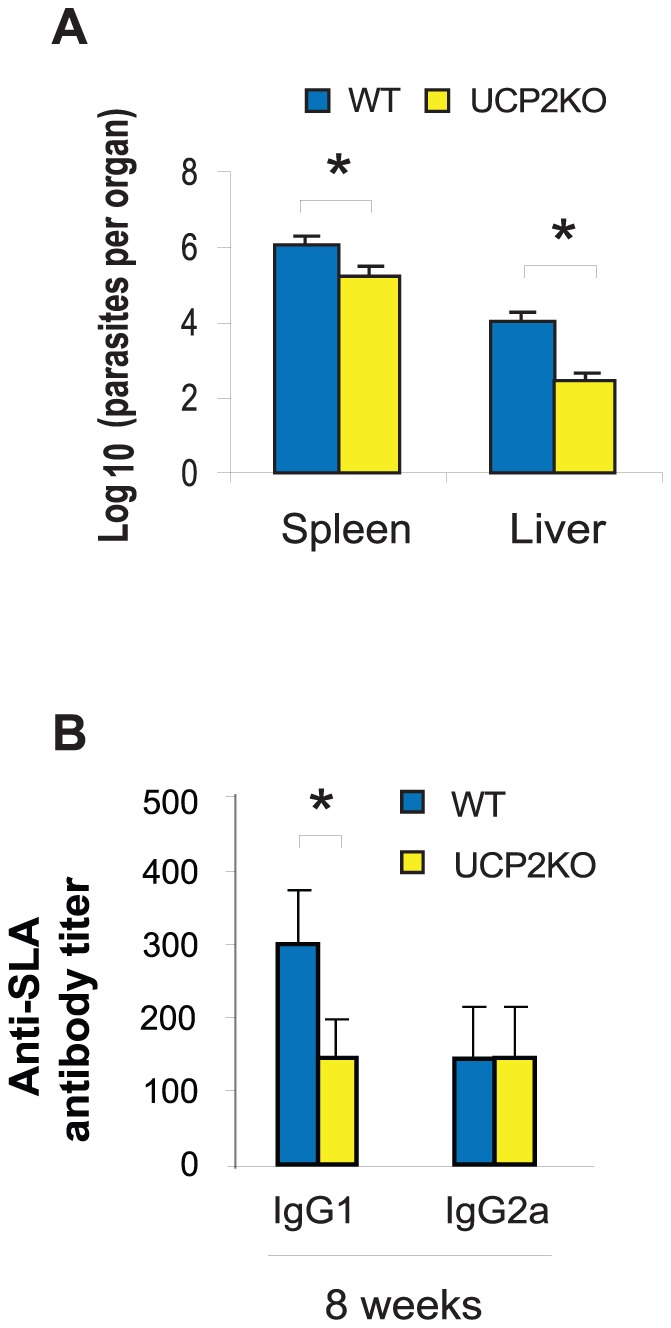
Outcome of visceral infection in UCP2KO and WT C57BL/6 mice. All mice were challenged intravenously with 5×10^5^ live, stationary-phase *L. infantum* parasites, and spleens, livers and sera were harvested at 8 weeks after infection. A. UCP2KO mice reduce parasite loads (assessed by limiting dilution) in spleen and liver more efficiently than WT mice. B. Anti-SLA antibody (IgG1 and IgG2a) titers were determined by ELISA. Data are presented as mean ± S.D. (n = 6). Asterisks indicate *P*<0.05 with respect to WT mice.

To investigate the effects of host UCP2 deficiency on *L. infantum* systemic infection, we evaluated parasite loads in the spleen and liver by limiting dilution after 8 weeks of infection. Parasite loads in both organs were significantly smaller in infected UCP2KO mice than in infected WT mice (Fig. 4A).

### Humoral response and cytokine production in UCP2KO and WT C57BL/6 mice infected with *L. infantum*

Anti-SLA IgG1 antibody levels were significantly lower in UCP2KO mice than in WT mice after 4 and 12 weeks of infection. However, IgG2a antibody levels were similar between both groups of mice (Fig. 4B). To examine whether host UCP2 deficiency might play another role in the VL model, we evaluated the pattern of cytokine production in the spleen in both groups of mice. Spleens of UCP2KO mice showed significantly higher levels of IFN-γ and IL-13 than the spleens of WT mice (Table 4). However, IL-10 levels were similar between the two groups of mice.

**Table 4:** Cytokine production in mice infected with *L. infantum* at 8 weeks after infection.

pg/ml			
	IFN-γ	IL- 13	IL- 10
**WT**	552±100	241±20	69±20
**UCP2KO**	796±100 (*)	325±20 (*)	82±20

Data are presented as mean ± S.D. (n = 6). Asterisks indicate *P*<0.05 with respect to WT mice.

## Discussion

Since mitochondrial UCP2 has been proposed to negatively regulate ROS levels and thereby help protect against oxidative damage, we have decided to investigate the ability to mount responses against *Leishmania* parasites in macrophages from UCP2KO and WT mice. Protozoan parasites of the genus *Leishmania* reside and replicate predominantly within macrophages, cells involved in the innate immune response. It has recently been suggested that *Leishmania* upregulates UCP2 after infection in order to decrease host ROS levels and thereby suppress macrophage defense machinery [9].

In agreement with previous studies using *L. pifanoi* [32], a member of the *Leishmania mexicana* complex causing CL in the New World [26], we consider that infection of macrophages with axenically cultured *L. pifanoi* amastigotes represent a suitable *in vitro* model to study mechanisms of phagocytic uptake, killing activity and ROS production in mammalian cells. In laboratory experiments (unpublished data), we found no significant differences in the early phagocytosis, 2 h and 4 h after infection with *L. pifanoi*, between UCP2KO and WT peritoneal macrophages. Nevertheless, we demonstrate here an enhanced resistance (Killing activity at 24 h) of UCP2-deficient macrophages to infection with the intracellular protozoan *L. pifanoi*. In addition to intracellular killing of parasites, we assessed ROS production by peritoneal macrophages infected with *L. pifanoi* amastigotes, the intracellular form of the mammalian stage of the parasite life. These results are of great mechanistic importance since the activity of UCP2 should lower superoxide formation at the mitochondrial respiratory chain [4]. Therefore, it suggests that mitochondrial ROS formation is an important contributor to resistance of UCP2KO mice to *L. pifanoi* infection and it would be in line with the reported resistance to infection with *Toxoplasma gondii* [8]. We hypothesize that our results reflect that natural synergy between increased ROS production and increased nitrite production was greatly enhanced in macrophages from UCP2KO mice. Several studies have addressed the role of ROS and NO in killing of *Leishmania* parasites [33]–[35].

Our present work showed that thioglycollate-elicited macrophages population from peritoneum of both WT and UCP2KO mice, stimulated by IFN-gamma and LPS, produced IL-12 p40 independently of *Leishmania* infection. Furthermore, none of the three *Leishmania* species analyzed in our study (*L. pifanoi*, *L. major* or *L. infantum*) was able to inhibit IL-12 p40 production by peritoneal macrophages during the early stage of infection (24 h), as characterized in previous studies [36]. The early induction and maintenance of IL-12-dependent IFN-gamma production is critical to the host resistance to *Leishmania* infection [36], [37]. The IFN-γ induced production of NO is not only crucial for the direct leishmanicidal activity of macrophages but also triggers the IL-12 signaling cascade that leads to further IFN-γ production by natural killer cells [38]. Regarding IL-12 production, it has been suggested that *Leishmania* parasites of different species are potent inhibitors of macrophage IL-12 production [29], [39], [40]. However, the ability of macrophages to produce IL-12 seems to be related to their maturation stage [36], [41]. Also, this capacity varies depending on the stage of *Leishmania* infection [36]. We found NO, a principal effector molecule that mediates the intracellular killing of *Leishmania* parasites [42], to be present at higher levels in UCP2KO peritoneal macrophages than in WT ones after infection with any of three *Leishmania* species. Therefore, our study provides *in vitro* evidence that macrophages from UCP2KO mice have developed several mechanisms to combat intracellular *Leishmania* pathogens more efficiently. These include not only maintenance of IL-12 p40 production throughout infection that is required to prevent the loss of Th1 cells [37], but also significant high amounts of ROS and NO after *Leishmania* infection.

Subsequently we found that UCP2KO C57BL/6 mice mounted a stronger host defense against *L. major* and *L. infantum* infections *in vivo* than did the corresponding WT mice. In contrast to BALB/c mice, C57BL/6 mice after *L. major* infection develop self-healing cutaneous lesions and control parasite multiplication [43]. It has been reported that CL in C57BL/6 mice occurs in two phases following infection in the ear: a remarkably silent phase lasting about 4 weeks favors parasite amplification in the dermis without the formation of a lesion, followed by the development of a cutaneous lesion that coincides with a reduction in parasite load at the site of infection [44]. In our study mice were inoculated in the footpad and the evolution of infection occurred in the two mentioned phases above. UCP2KO C57BL/6 mice were significantly better than their WT counterparts at controlling *L. major*: after 6 weeks of infection, the UCP2KO mice showed significantly smaller footpad swelling and lower parasite burden. Nevertheless, the lesions in the UCP2KO mice resolved at the same time as those in WT mice, suggesting that lack of UCP2 accelerated the development of an adaptive immune response at the level of parasite load but not at the level of lesion resolution. This may reflect the intrinsic features of the resistant C57BL/6 strain, which, like humans, develops self-healing skin lesions following *L. major* infection [45], [46]. Taken together, our data showed that UCP2 deficiency was dispensable for the resolution of the lesions at the site of infection, but was advantageous to achieve an early reduction of parasite load in the DLN and was essential for efficient parasite clearance in the spleen after 12 weeks p.i. These results are consistent with previous research showing that the capacity to kill intracellular *L. major* parasites was dramatically reduced in mice deficient in the production of reactive oxygen intermediates (ROI) [34]. In addition, it is well established that NO acts as a leishmanicidal molecule [42], interfering with arginase activity in infected host cells and subsequently with cellular metabolism of *Leishmania*. Inhibition of arginase activity during infection has a clear therapeutic effect, as evidenced by markedly reduced pathology and efficient control of parasite replication [47]. In the present study, arginase activity was lower and leishmanicidal nitrite levels were higher in phagocytic cells from UCP2KO mice than in such cells from WT mice. This reflects greater intracellular killing of *Leishmania* parasites in UCP2KO mice.

Because susceptibility to *Leishmania* infection correlates with the development of CD4^+^ Th2 cells, whereas resistance correlates with the development of CD4^+^ Th1 cells [43], we compared the production of the cytokines IFN-γ, IL-17 and IL-4 in DLNs from *L. major*-infected UCP2KO and WT mice after *in vitro* stimulation with SLA-pulsed DCs. We found that infected UCP2KO mice showed a higher Th1 and Th17 anti-*Leishmania* immune response and a weaker Th2 response than did WT mice. Consistent with our findings, UCP2KO mice infected with another intracellular pathogen, *Listeria monocytogenes*, showed high levels of ROS in the spleen, and this was associated with a switch away from anti-inflammatory cytokine production toward proinflammatory cytokine production, as well as with a significant increase in recruited splenic phagocytes [7]. We further found UCP2KO mice to have elevated IL-17 levels, which is consistent with previous reports that acquisition of a resistant phenotype in both VL and CL murine models is related to Th1- and Th17-mediated, parasite-specific cellular responses [48], [49].

In addition, we assessed the relative production of IgG2a and IgG1 isotypes, since these have been widely used as markers, respectively, of the induction of Th1- and Th2-type immune responses [50]. Indeed, anti-*Leishmania* IgG1 antibody production fails to protect against this intracellular pathogen and contributes to disease progression [31]. In accordance with the notion that UCP2KO mice are highly resistant to *Leishmania* infection, we found not only higher levels of *Leishmania*-specific IgG1 antibodies in WT mice than UCP2-deficient ones, but also higher IL-4 levels in DLNs from WT mice. Th2 IL-4 cytokine has been reported to induce antibody production [30]. Taken together, these data support our hypothesis that UCP2KO mice have developed increased resistance to *L. major* that led to a faster resolution of the infection.

The visceral form of the disease (VL) was also evaluated in order to investigate the role of UCP2 in host immune defense. C57BL/6 and BALB/c strains of mice susceptible to *L. infantum* infection possess a non-functional *Slc11a1* gene, preventing them from controlling early parasite growth in the liver [21], [51], [52]. However, during later stages of *L. infantum* infection, these strains develop mature granulomas that can eliminate the parasites in the liver [53], [54]. It has been reported that while ROS are not crucial for the activation of leishmanicidal abilities of hepatic mature granulomas during later stages of VL, NO and ROS do contribute to parasite control [33]. A recent study of murine VL reported that granuloma maturation is delayed in mice deficient in IL-13 due to defective IFN-γ production and elevated IL-4 and IL-10 levels [55]. That study suggested that IL-13 plays a crucial role in ensuring efficient hepatic granuloma maturation to control parasite load during VL [55]. Therefore we evaluated the pattern of cytokine production by the spleen in UCP2KO and WT mice in the present study. Our finding that IFN-γ and IL-13 levels in the spleen were significantly higher in UCP2KO mice supports the notion that they are better able to control *L. infantum* infection. This is consistent with a recent study in which siRNA-mediated silencing of the *UCP2* gene in a mouse model of VL stimulated mitochondrial ROS production, which induced a proinflammatory cytokine response and subsequently reduced parasite burden [9]. Also, anti-*Leishmania* IgG1 antibody production, associated with increased susceptibility to the disease as mentioned above, was significant higher in WT mice as compared to UCP2-deficient ones.

In conclusion, our study for the first time provided *in vivo* evidence that UCP2 deficiency was beneficial to achieve an early control of parasite growth in the DLN and was critical to avoid visceralization of the parasite during CL. Also, it does contribute to parasite control in murine model of VL. The role of UCP2 as part of the cellular anti-oxidant defence is well established. Since alterations in UCP2 levels are involved in a number of pathologies, this protein is currently considered as a drug target [2]. A good example comes from the cancer field and, thus, it has been shown that UCP2 levels are elevated in drug-resistant tumour cells and that UCP2 inhibitors sensitize cancer cells to chemotherapeutic agents [3]. In our work, we demonstrate that knock-out of the UCP2 gene allows mice to respond better to CL and VL. Therefore, it can be envisaged that UCP2 inhibitors may improve efficacy of combined therapies against leishmaniosis.
